# The involvement of high mobility group 1 cytokine and phospholipases A2 in diabetic retinopathy

**DOI:** 10.1186/1476-511X-13-156

**Published:** 2014-10-08

**Authors:** Yan Gong, Xin Jin, Quan-Shun Wang, Shi-Hui Wei, Bao-Ke Hou, Hong-Yang Li, Mao-Nian Zhang, Zhao-Hui Li

**Affiliations:** Department of Ophthalmology, Chinese PLA General Hospital, No. 28 Fuxing Road, Haidian District, Beijing, 100853 China; Department of Health Care, Headquarters Clinic of Chinese People’s Armed Police Force, Beijing, China

**Keywords:** Blood retinal barrier, Micro vessels, Retinal pericytes, Endothelial cells

## Abstract

**Background:**

Diabetic retinopathy, the main microvascular complications of diabetes and one of the leading causes of blindness worldwide. Interesting reports on the role of inflammatory/proangiogenic high mobility group 1 (HMGB-1) cytokine and phospholipases A2 (PLA2) in neovascularization have diverted our concentration to reveal whether HMGB-1 and PLA2 plays role in diabetic retinopathy.

**Methods:**

We performed our study in streptozotocin (STZ)-induced diabetic rat model. The expression levels of the cytokines, chemokines, and cell adhesion molecules in retinal tissues were evaluated by quantitative RT-PCR. HMGB-1 and PLA2 protein levels along with VEGF, TNF-α, IL-1β and ICAM-1 levels were also measured.

**Results:**

We observed the retinal pericytes, endothelial injury/death and breakdown of blood–retinal barrier (BRB). The protein expression of HMGB-1, PLA2 and IL-1β were significantly increased in micro vessels from retina of diabetic rats. Diabetic rats had also high retinal levels of VEGF, ICAM-1 and TNF-α. Further investigation revealed that pericyte death is mediated by HMGB-1-induced cytotoxic activity of glial cells, while HMGB-1 can directly mediate endothelial cell death. Similarly, increased expression of PLA2 represents the diabetic mediated alteration of BRB, perhaps up regulating the VEGF.

**Conclusions:**

Our data suggest that HMGB-1 and PLA2 involved in retinal pericyte and endothelial injury and cell death in diabetic retinopathy. From this study, we suggest that HMGB-1 and PLA2 may be interesting targets in managing diabetic retinopathy.

## Introduction

Diabetic retinopathy is the most common micro-vascular complication of diabetes and remains one of the leading causes of blindness in adults [1]. As a global concern, diabetes affects more than 360 million individuals worldwide. This number is expected to exceed half a billion by 2030 [2]. About one in three individuals with diabetes has signs of retinopathy, with in these, one-third may have diabetic macular edema (DME) or proliferative diabetic retinopathy (PDR), two vision-threatening forms of diabetic retinopathy [3]. Diabetic retinopathy is a progressive alteration in the retinal microvasculature, leading to areas of retinal non-perfusion, increased vasopermeability, and pathologic intraocular proliferation of retinal vessels in response to retinal nonperfusion. Due to progressive retinal capillary dropout, the ischemic retina mounts an angiogenic response leading to a more advanced form of the disease, proliferative diabetic retinopathy [1]. However, the mechanism behind was not clear.

HMGB-1 protein is a nuclear DNA binding protein released passively from necrotic cells as well as actively from monocytes/macrophages and endothelial cells. HMGB-1 can activate the pattern recognition receptors toll-like receptor 4 (TLR4) and receptor for advanced glycation end products (RAGE), triggering inflammation and damage as well as promoting angiogenesis in tissue [4, 5]. Studies have reported that noxious stimuli such as amyloid beta induce activation of cytosolic PLA2 in bovine pericytes [6, 7] and, recent study shown have that cytosolic PLA2 activation is required for hypoxia-induced VEGF-dependent retinal neovascularization [8].

Numerous biochemical changes have been observed in the vascular tissue of the retina, which are believed to be involved in diabetic retinopathy. A major change considers the signaling of vascular endothelial growth factor (VEGF), the crucial regulator of vasculogenesis, angiogenesis, lymphangiogenesis and vascular permeability in vertebrates [9]. There is also increasing evidence that inflammation has a key role in the pathogenesis of diabetic retinopathy, which is characterized by early breakdown of BRB and loss of pericytes/endothelial cells, which are essential for retinal capillary structure and function [3, 10]. Vascular adhesion molecules such as intercellular adhesion molecule-1 (ICAM-1) and cytokines such as TNF-α, among many others, have been implicated in the pathogenesis of DR [11]. VEGF increases retinal vascular expression of ICAM-1 [11], and this latter is directly involved in inflammation through its interaction with different cytokines such as TNF-α [12]. Based on the previous reports, our present study is aimed to reveal the possible mechanism of HMGB-1 and PLA2 on their involvement in diabetic retinopathy. The study was performed in streptozotocin (STZ)- induced diabetic rat model.

## Material and methods

### Reagents

All chemicals and reagents were purchased from Sigma-Aldrich (St. Louis, MO), Life Technologies (Grand Island, NY), and Thermo Scientific (Rockford, IL), unless otherwise indicated. Recombinant HMGB-1 was purchased from R&D Systems (Minneapolis, MN) and IBL International Corp (Toronto, ON). Rabbit polyclonal antibody against von Willebrand factor, mouse monoclonal antibodies against cPLA2, α-actin and GAPDH were purchased from Santa Cruz (Santa Cruz, CA). Streptozotocin (STZ) was purchased from Sigma. All the other reagents were obtained from standard commercial suppliers unless otherwise noted.

### Experimental animals

Male Sprague–Dawley rats weighing approximately 200 g were used for the experiment. All the animals were treated according to the Association for Research in Vision and Ophthalmology Statement for the Use of Animals in Ophthalmic and Vision Research. Animals were housed under standard conditions of temperature and humidity, with a 12 hour light/dark cycle and free access to food and water. After 12 h of fasting, the animals received a single 60 mg/kg intravenous (i.v.) injection of STZ in 10 mM sodium citrate buffer, pH 4.5 (1 ml/kg dose volume). Control (non-diabetic) animals were fasted and received citrate buffer alone. After 24 h, animals with blood glucose levels > 250 mg/dl were considered diabetic, and randomly divided in groups of ten animals each. The diabetic state was confirmed by evaluating glycemia daily through a blood glucose meter (Accu-Check Active1, Roche Diagnostic, Milan, Italy). Body weight was assessed daily. AACOCF3 was dissolved in saline and injected intravitreally (ITV; 2 ml) at 0.1, 1, 10 and 50 mM. All the experiments were performed 15 days following the induction of diabetes.

### Cell cultures

Primary microvascular endothelial cells from bovine retinas (BREC) were purchased from European Collection of Cell Cultures (ECACC) and were fed with Ham’s F-10 medium as previously described [13]. Primary microvascular pericytes (BRPC) were isolated from bovine retina microvessels as previously described [14]. The isolated cells were then cultured in DMEM supplemented with 10% Fetal Bovine Serum, 100 U/ml penicillin, and 100 mg/ml streptomycin. Morphological changes and cell viability was determined by MTT test.

### TUNEL assay

The terminal deoxynucleotidyl transferase-mediated biotinylated UTP nick end labeling (TUNEL) reaction was done along with DAPI staining. The protocol was adapted from Gavrieli et al. [15]. The prepared tissue sections were deparaffinized in xylene for 10 min and hydrated through a graded ethanol series. The TUNEL assay was then carried out according to the manufacturer’s (Roche Diagnostics, Meylan, France) instructions; the samples were stained with DAPI solution after the TUNEL reaction.

### Evaluation of protein expression

Rat eyes were collected two weeks after STZ administration, and each retina was processed as previously described [16]. Briefly, the retina was homogenized in 100 ml of solution containing (mM): imidazole hydrochloride, 20; KCl, 100; MgCl, 1; EGTA, 1; NaF, 10; sodium molybdinate, 1; EDTA, 1; plus 1% Triton. The solution was supplemented with a cocktail of protease inhibitors (Complete Protease Inhibitor Cocktail; Roche, Basel, Switzerland) before use. The soluble proteins, 10–20 μg, were loaded and separated by 10% SDS–PAGE and blotted onto a polyvinylidene fluoride membrane. Then, the membranes were blocked with 5% nonfat dry milk powder solution for 1 h at room temperature before an overnight incubation with primary antibodies TNF-α, VEGF, IL-1β and ICAM-1 (Santa Cruz Biotech.) at 4°C. After rinsing the membranes, they were incubated with secondary antibody for 1 h at room temperature and the bands were made more visible by enhanced chemiluminescence as described elsewhere [17]. The intensity of each band was normalized to that of β-actin and quantified using the Image J analysis.

### Gene expression analysis

Quantitative RT-PCR was performed to analysis the gene expression status by using gene-specific primers (Table 1). Specifically, RNA was extracted from retinal cells using RNA Nanoprep kit (Agilent Technologies, Santa Clara, CA), and reverse transcribed with the Reverse Transcription System (Promega, Madison, WI) to synthesize cDNA. Quantitative PCR was performed in the Rotor-Gene Q Cycler (Qiagen, Valencia, CA) using the SYBR GREEN PCR Master Mix (Qiagen, Valencia, CA). For each gene, relative expression was calculated by comparison with a standard curve, following normalization to the housekeeping gene β-actin expression chosen as a control.

**Table 1:** **List of RT-PCR primers**

Gene	Forward	Reverse
β-Actin	5′-CGT AAA GAC CTC TAT GCC AA-3′	5′-AGC CAT GCC AAA TGT GTC AT-3′
TNF-α	5′-CAA AAT TCG AGT GAC AAG CCT G-3′	5′-GAG ATC CAT GCC GTT GGC-3′
VEGF	5′-AAC CAT GAA CTT TCT GCT CTC TTG-3′	5′-GCC TGG CTC ACC GCCTTG GCT TGT C-3′
ICAM-1	5′-CCC TGT CAG TCC GGA AAT AA-3′	5′-GAT GAC TTT TGA GGG GGA CA-3′
PLA2	5′-CAG CAA GGA UCC UCG CUA U-3′	5′-CAG AAU GCU UCC AAU CGU A-3′

### Statistical analysis

Statistical significance between two groups was analyzed by Student’s t-test. One-way analysis of variance (ANOVA), followed by Tukey’s post hoc test, was used to compare the means for the multiple groups. The p value < 0.05 was considered statistically significant.

## Results

### Changes observed at the end of experiments

The diabetes was induced after twelve weeks of STZ injection; we found a significant (p < 0.05) difference between the normal and diabetic rats in body weight, eyeball weight, and serum glucose levels. The intakes of water and food were markedly increased in the diabetic rats (Table 2).

**Table 2:** **Basic Parameters measured at the end of experiment**

Parameters	Normal (n=12)	STZ-induced diabetic rats (n=12)
Body weight (g)	425.1 ± 4.9	214.4 ± 11.4
Eyeball weight (mg/100 g B.W)	39.8 ± 4.5	74.3 ± 12.9
Serum glucose (mg/dL)	135.13 ± 3.12	540.02 ± 37.51

### Apoptosis in the retinal ganglion cell layer in STZ-induced diabetic rats

**Figure 1:**
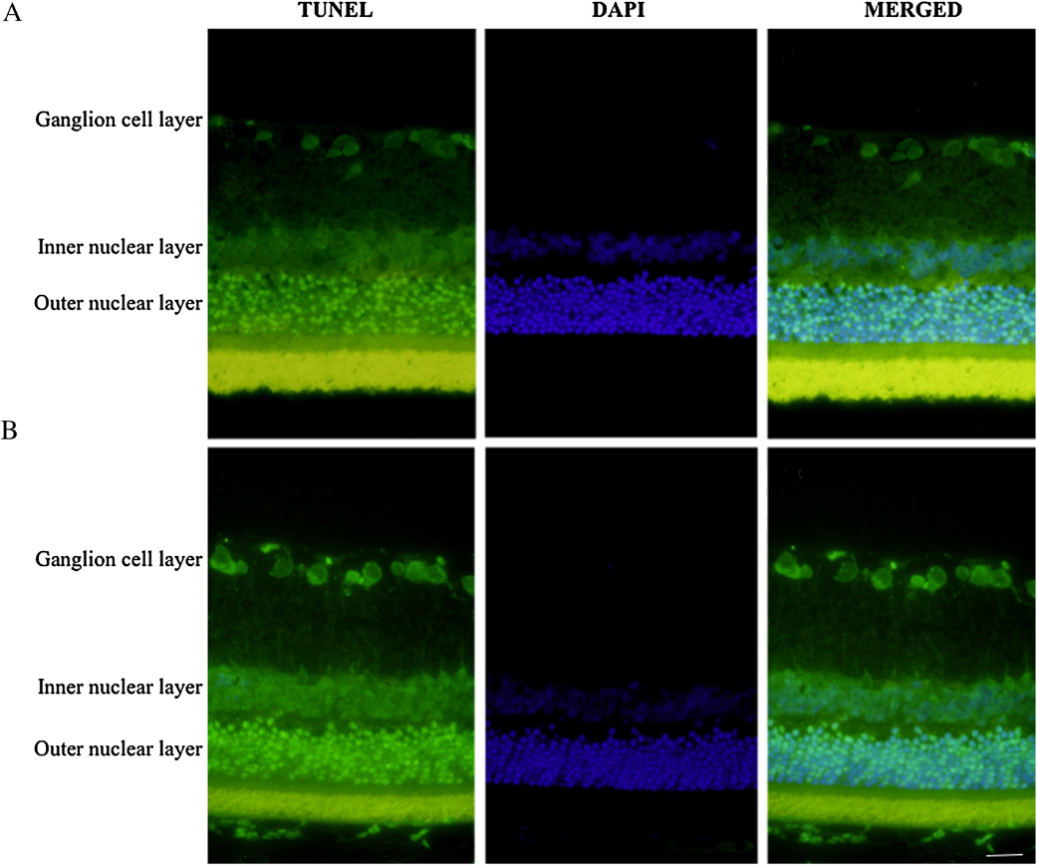
**Apoptosis detected in retinal cell by TUNEL and DAPI staining. A**. Represent the retinal layer of control rats. **B**. Represents the retinal layer of STZ-induced diabetic retinopathy rats. The apoptotic retinal ganglion cell death was detected by TUNEL (green), and the nuclei (blue) were stained with DAPI. Scale bar = 50 μm.

To identify apoptotic cell death in the retina affected by diabetes, whole retinas were reacted with TUNEL. The TUNEL positive cells were stained with green, DAPI with blue and the merged image shows the combination of green and blue (Figure 1). Twelve weeks after the induction of diabetes, TUNEL-positive cells were observed in the ganglion cell layer, and a large number of apoptotic ganglion cells were present in the retinas of STZ-induced diabetic rats (Figure 1).

### Evaluation of mRNA levels in retinal tissue

**Figure 2:**
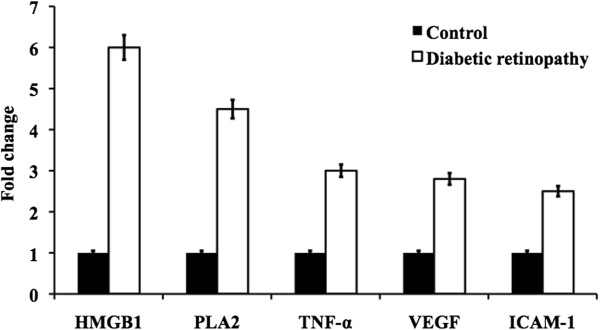
**Represent the mRNA expression of HMGB1, PLA2, TNF-α, VEGF, ICAM-1 from the retinal tissue of control and diabetic retinopathy rats.**

The mRNA levels of the cytokines, chemokines, and cell adhesion molecules in retinal tissues were evaluated by quantitative RT-PCR. The samples from diabetic retinopathy (DR) and their respective control were observed. The mRNA levels of HMGB1 and PLA2 were found a significant (4.5 fold) increase in DR tissue (Figure 2). Similarly, TNF-α, VEGF and ICAM-1 were increased at least to 2.5 fold in DR when compared to that of control tissue.

### Evaluation of protein levels in retinal tissue

**Figure 3:**
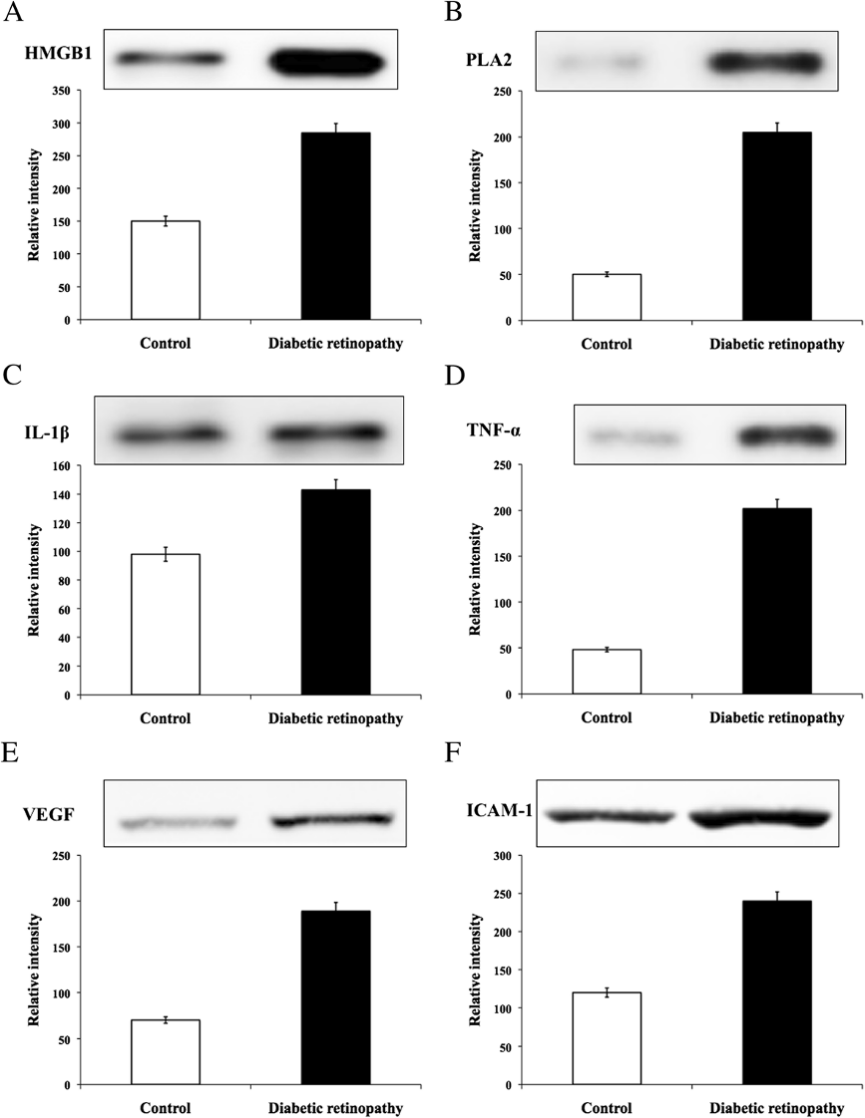
**Represent the western blot detecting the protein levels of A. HMGB1, B. PLA2, C. IL-1β, D. TNF-α, E. VEGF, and F. ICAM-1 with the quantification of their corresponding band intensity from the retinal tissue of control and diabetic retinopathy rats.**

The protein levels were detected and quantified by using western blot analysis in retinal tissues of DR and their respective control. Interestingly, similar to the results obtained in the gene expression study. We observed significantly elevated level of HMGB1 and PLA2 in DR tissue than that of normal tissue (Figure 3). The protein levels of IL-1β, TNF-α, VEGF and ICAM-1 were found similar elevation in DR tissue when compared to the control tissue. The corresponding band intensity was plotted in bar graph (Figure 3). Interestingly, HMGB1 was more prominently found among others proteins evaluated in both gene expression as well as protein study.

### Evaluation of PLA2 activity in microvessels of retina from control and diabetic rats

**Figure 4:**
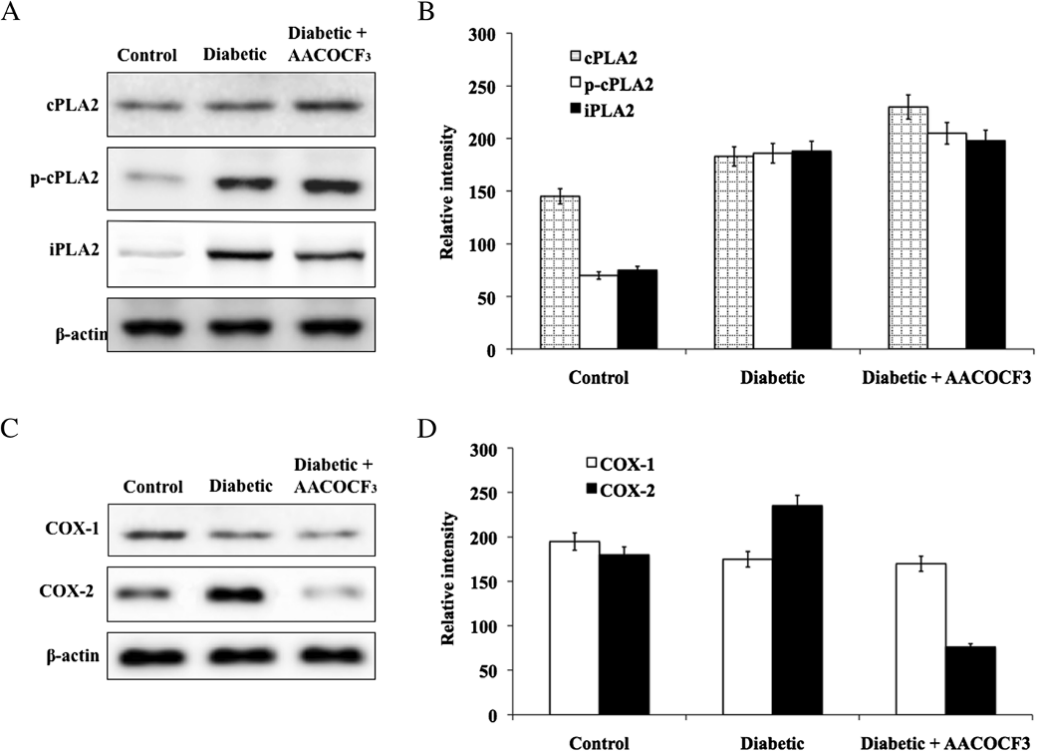
**Evaluation of PLA2 and COX proteins. A**. Represent the protein expression of total protein cPLA2 and its phosphorylated form in control and diabetic retinopathy rats and **B**. Represent the quantification of corresponding band intensity using image-J analysis. **C**. Represent the protein expression of COX-1 and COX-2 in control and diabetic retinopathy rats. **D**. Represent the quantification of COX-1 and COX-2 band intensity using image-J analysis.

PLA2 activity was strongly increased in microvessels from DR rats for about 3 fold when compared to control rats (Table 3). Treatment with AACOCF3 (ITV, 2 ml, 50 mM) reduced PLA2 activity down to the level of control rats. The western blot analysis was performed to evaluate the expression of cPLA2 and its phosphorylated form (p-cPLA2), iPLA2, COX-1 and COX-2 in microvessel lysates obtained from retina of experimental rats (Figure 4). An increased expression of cPLA2 was found in microvessels from retina of diabetic rats (Figure 4). The phosphorylated form of cPLA2 was further increased (about 9.5 fold) in diabetic rats and its expression did not change after treatment with AACOCF3. Moreover, iPLA2 expression was increased for about 2.6 fold in diabetic rats and, as expected, its expression did not change after treatment with AACOCF3. These results are consistent with in vitro findings (data not shown) showing in endothelial cells a contribution of both cPLA2 and iPLA2 to the increase in total PLA2 activity, after the induction of diabetes.

**Table 3:** **PLA2 activity**

Groups	PLA2 activity (pmol/min/mg)
Control (non-diabetic)	14.7 ± 1.1
Diabetic (STZ)	55.9 ± 5.7*
Diabetic + AACOCF3	21.2 ± 2.4**

*Indicate the significance (p < 0.05) Diabetic Vs Control.

**Indicate the significance (p < 0.05) Diabetic Vs Diabetic + AACOCF3.

### Immunohistochemistry of retinal tissue for HMGB1 staining

**Figure 5:**
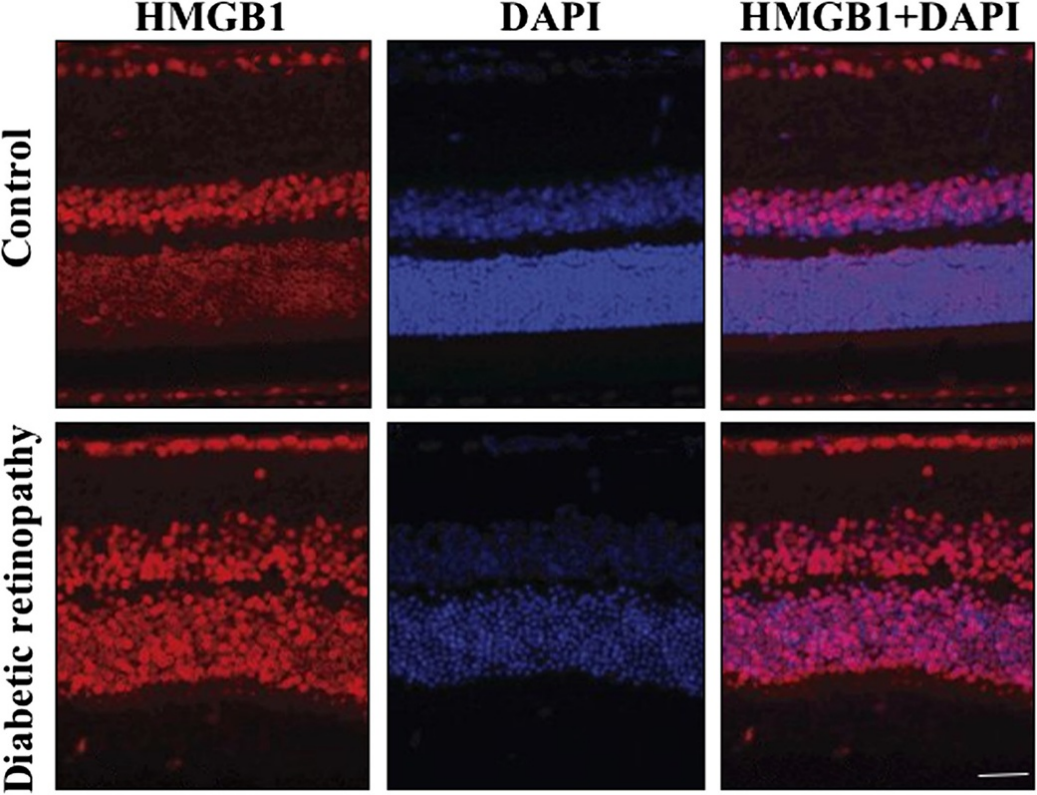
**Represent the HMGB1 (red) and DAPI (blue) staining of retinal layer of control and STZ-induced diabetic retinopathy rats.** The high expression of HMGB1 was noted in diabetic retinopathy. Scale bar = 50 μm.

The immunohistochemical study was performed to determine the presence of HMGB1 and to quantify the staining intensity in retinal tissues of DR and their respective control. The HMGB1 stained with red, the DAPI stained with blue color and the merged image was also presented with the combination of red and blue (Figure 5). The abundant HMGB1 expression was observed in DR tissues when compared to the control tissue. This result indicates the significant involvement of HMGB1 in DR.

## Discussion

Diabetic retinopathy is one of the major microvascular complications of diabetes and one of the most common causes of blindness among elderly people [1, 18]. During diabetes the retinal microvessel cells are subjected to the action of prolonged hyperglycemia, and undergo progressive dysfunction leading to neovascularization (NV) and vascular leakage [19]. The histopathology of diabetic retinopathy include loss of blood-vessel pericytes and endothelial cells, and abnormal new blood vessel growth on the surface of the retina in the more advanced stage of the disease, proliferative diabetic retinopathy [1, 18]. Recent reports have elucidated that HMGB-1 is involved in the pathogenesis of diabetic microvascular complications, including diabetic retinopathy [20, 21]. However, cellular mechanisms of HMGB-1 action in the diabetic retina remain incompletely understood. HMGB-1, a DNA-binding nuclear protein can be released actively after cytokine stimulation and passively during necrotic cell death [4]. HMGB-1 can promote inflammatory responses and cause damage by numerous mechanisms [4, 22]. It was also shown that HMGB-1 could promote angiogenesis in some tissues [5, 23]. In this study, we demonstrated that HMGB-1 could mediate endothelial cell death directly, while pericyte death was indirectly mediated by HMGB-1-induced cytotoxic activity of glial cells. HMGB-1 can also affect endothelial cell activity. However, our findings suggest that HMGB-1 plays an insignificant role in retina and choroidal neovascularization.

Similarly, several isoforms of PLA2 have been identified and localized in different structures of the eye [24]. They have also been associated with the regulation of retinal pigment epithelium phagocytosis and of photoreceptor cell renewal [25] or to several retinal disease mechanisms such as retinal edema formation in diabetic rats [26] or in retinal angiogenesis in a rodent model of retinopathy of prematurity [27]. Inhibition of PLA2, using either a iPLA2-specific compound or cPLA2/iPLA2 non-selective compound, dampened the glucose-induced elevation of PGE2 and VEGF both in BREC and BRPC. Furthermore, cPLA2 and iPLA2 siRNA transfection decreased VEGF-induced BREC proliferation and BRPC loss, suggesting the importance of cPLA2 as a positive regulator of VEGF-induced retinal angiogenesis. This knock down experiment further validates the results obtained with inhibitors, that may otherwise be theoretically attributed, at least in part, to nonspecific, off target effects [28].

The gene expression study had revealed that HMGB-1 and PLA2 affects endothelial cell activity. We found a significant increased expression of HMGB-1 and PLA2, similar pattern was also found in the expression of TNF-α, VEGF and ICAM-1 in diabetic retinal cells. HMGB-1-dependent increased expression of chemokines by glial cells and endothelial cells can attract leukocytes to the diabetic retina [29]. Increased expression of ICAM-1 can facilitate increased adhesion of leukocytes to endothelia [29]. It was shown that adherent leukocytes mediate endothelial cell injury and death in the diabetic retina [29]. These data suggest that HMGB-1 and PLA2 could also indirectly mediate endothelial cell death in patients suffering from diabetic retinopathy. In addition, these results were supported by the protein analysis of TNF-α, VEGF, IL-1β and ICAM-1 in the diabetic retinal tissues.

Immunohistochemical analysis of human eyes has shown constitutive expression of VEGFR-1, but not of VEGFR-2, in samples from healthy controls, and up-regulation of VEGFR-1 and new expression of VEGFR-2 in samples from diabetic patients [30]. It is well known that VEGF directly stimulates EC proliferation and migration, which leads to the formation of new vessels; however, it seems also that, in some circumstances, VEGF may act as an inhibitor of NV by disrupting vascular smooth muscle cells function; specifically, VEGF ablates PC coverage of nascent vascular sprouts leading to vessel destabilization [31]. In the present study, our result shows that high glucose or diabetes increases VEGF but exert opposite effects on EC and PC cell number are in accordance with this assumption. Blood vessels become hemorrhagic and hyperdilated when they lose pericytes, the vessel support cells [32]. The loss of the pericytes is characteristic for the diabetic retina [1, 18]. The published literature demonstrated elevated levels of HMGB-1 in diabetic retinas [20, 21].

In this study, we have investigated the cellular mechanism of action of HMGB-1 and PLA2 in diabetic retina. Our findings indicate that retinal pericyte and endothelial injury and death in diabetic retinopathy might be due to HMGB-1/PLA2 induced cytotoxic activity of glial cells as well as the direct effect of HMGB-1 on endothelial cells. At the same time, our data suggest that HMGB-1/PLA2 plays an insignificant role in retinal and micro-neovascularization. Thus, this study provides further insight into the research on drug target or therapeutic role of HMGB-1/PLA2 in diabetic retinopathy.
